# Neoadjuvant tyrosine kinase inhibitors in rectal gastrointestinal stromal tumours: a provision for enhanced oncological and functional outcomes

**DOI:** 10.1007/s10147-021-01867-2

**Published:** 2021-02-02

**Authors:** Zachary Zihui Yong, Jolene Si Min Wong, Melissa Ching Ching Teo, Claramae Shulyn Chia, Chin-Ann Johnny Ong, Mohamad Farid, Grace Hwei Ching Tan

**Affiliations:** 1 Department of Sarcoma, Peritoneal and Rare Tumours (SPRinT), Division of Surgery and Surgical Oncology, National Cancer Centre Singapore, 11 Hospital Crescent, Singapore, 169610 Singapore; 2 SingHealth Duke-NUS Oncology Academic Clinical Program, Duke-NUS Medical School, 8 College Road, Singapore, 169857 Singapore; 3 Laboratory of Applied Human Genetics, Division of Medical Sciences, National Cancer Centre Singapore, 11 Hospital Crescent, Singapore, 169610 Singapore; 4 Institute of Molecular and Cell Biology, A*STAR Research Entities, 61 Biopolis Drive, Singapore, 138673 Singapore; 5 Division of Medical Oncology, National Cancer Centre Singapore, 11 Hospital Crescent, Singapore, 169610 Singapore

**Keywords:** Gastrointestinal stromal tumours, Tyrosine kinase inhibitors, Neoadjuvant treatment

## Abstract

**Background:**

The role of tyrosine kinase inhibitors (TKI) in the neoadjuvant setting and the optimal duration of therapy remains poorly defined. As such, we aim to evaluate the impact of neoadjuvant TKI on oncological and functional outcomes in our cohort of patients with rectal GISTs.

**Methods:**

A retrospective analysis of 36 consecutive patients who underwent treatment for rectal GIST at the National Cancer Centre Singapore from February 1996 to October 2017 was analysed. Surgical, recurrence and survival outcomes between the groups who underwent neoadjuvant therapy and those who underwent upfront surgery were compared.

**Results:**

Patients who received neoadjuvant treatment had significantly larger tumours (median size 7.1 vs. 6.0 cm, *p* = 0.04) and lower mitotic count (> 10 per 50 HPF, 14 vs. 70%, *p* = 0.03) when compared with the non-neoadjuvant group. With TKI pre-treatment (median duration 8.8 months), majority of patients (82%) achieved at least partial response to the therapy coupled with a significant downsizing effect of up to 39% (median size of 7.1–3.6 cm), resulting in similar rates of sphincter-sparing surgery (75 vs. 76%, *p* = 0.94) when compared with the non-neoadjuvant group. In general, neoadjuvant group had lower rates of local recurrence (0 vs. 69%, *p* = 0.04) and higher overall survival (7.4 vs. 5.7 years, *p* = 0.03) as compared to the non-neoadjuvant group.

**Conclusions:**

Neoadjuvant TKI has the benefit of downsizing unresectable rectal GIST to benefit from sphincter-sparing procedure and also confers protection against local recurrence and improves overall survival.

## Introduction

Gastrointestinal stromal tumours (GISTs) belong to a spectrum of mesenchymal tumours that range from indolent tumours to malignant sarcomas. After acquiring a mutation in the tyrosine kinase receptor c-KIT (CD117), the interstitial cells of Cajal (ICC), which are located in the myenteric plexus in the gastrointestinal wall and serve as a pacemaker of the gut, lose normal growth control and result in tumour formation [1]. The most common site of GISTs is the stomach, while rectal GISTs are rare and occur in only 5% of patients.

Approximately half of the patients at first presentation are not eligible for curative surgical resection due to the presence of metastatic disease [2]. Even with R0 resection, one-third of the patients develop local recurrence illustrating the need for systemic therapy to manage this disease [3]. Imatinib is a KIT and PDGFR-α receptor tyrosine kinases inhibitor, which are pathophysiological drivers of GISTs. Imatinib occupies the ATP-binding site of tyrosine kinase receptor to prevent autophosphorylation leading to proliferation arrest and apoptosis. This leads to the morphological finding of extensive sclerosis and hyalinization with scattered shrunken tumour cells in the previous viable cells. Before the introduction of imatinib, median survival for patients with GIST ranged from 10 to 20 months [4]. With the advent of imatinib, median survival increased to 51–57 months [5]. Currently, imatinib is approved as a first-line systemic treatment for KIT-positive unresectable and/or metastatic GISTs and has revolutionized their treatment.

Unlike its gastric counterpart, rectal GISTs have the worst clinical outcomes and poorest prognosis [6]. Although small rectal GISTs are amenable to local excision with sphincter preservation, a larger tumour size may preclude the ability to perform a function preserving surgery. Reported response rates to neoadjuvant imatinib range from 20 to 80% [7]. In the anatomically challenging pelvis, such good response represents a good opportunity to downsize rectal GISTs thereby reducing the risk of intraoperative tumour rupture and surgical morbidity while improving sphincter–preservation rates. Several small retrospective series have also shown the effectiveness of preoperative imatinib in improving local disease-free survival (DFS), DFS and overall survival (OS) [8].

This study aims to find out the clinical profile of patients with rectal GIST in our local population and determine if neoadjuvant TKI prior to surgery improves the outcomes for this rare disease.

## Patient and methods

The data were retrospectively collected from a prospective database of patients who underwent treatment for rectal GIST at the National Cancer Centre Singapore from February 1996 to October 2017. The conduct of the study was approved by the SingHealth Centralized Institutional Review Board (CIRB 2018/3065). A total of 36 consecutive patients were accrued and analysed. Preoperative parameters such as patients’ age, gender, race and ECOG status were analyzed.

All patients had a histological diagnosis of GIST and underwent preoperative imaging with a Computed Tomography (CT) scan of the chest, abdomen and pelvis or a Positron Emission Tomography (PET)-CT scan. Patients with distal metastatic disease were excluded from the analysis. Primary tumour size, mitotic count, stage according to NIH-Fletcher risk classification, imaging and endoscopic findings were reviewed and discussed at a multidisciplinary tumour board meeting [9].

Reasons for the recommendation of neoadjuvant therapy were collated. Patients recommended for neoadjuvant therapy had unresectable tumours (i.e. gross invasion into vital structures) or borderline resectable tumours in close proximity to vital structures such as the anal sphincter. In addition, they had to be of Eastern Cooperative Oncology Group (ECOG) status 0 or 1 prior to receiving neoadjuvant therapy. The dose, duration and tumour response to neoadjuvant therapy were evaluated with the Response Evaluation Criteria in Solid Tumours (RECIST) criteria based on the CT scan performed after completion of neoadjuvant therapy [10]. Patients who underwent neoadjuvant therapy and experienced progressive disease or developed distal metastases were not offered curative surgery.

Patients who underwent upfront surgery without neoadjuvant therapy were labelled as the non-neoadjuvant group. There were patients from the non-neoadjuvant group who were offered but did not undergo surgery, due to lack of consent or defaulted surgery. The type of surgery, residual tumour (R) classification and surgical complications were tabulated. Duration and response to adjuvant imatinib, together with the DFS and OS, were also compared between the neoadjuvant and non-neoadjuvant group. DFS is defined as all GISTs cancer events including local, regional and distant relapse; second malignancies and deaths without recurrence were censored. OS is defined as all deaths including those without recurrence.

Categorical variables were analyzed using the chi-square test or Fisher’s exact test when cell sizes are small (< 5). Haldane’s approximation was utilized by adding a constant of 0.5 to each cell if any observed frequencies were 0. The two-sample *t* test was used for normally distributed continuous variables while the Mann–Whitney *U* test was used for non-parametric continuous variable. *p* < 0.05 was considered statistically significant. Statistical analysis was performed on SPSS version 19.0.

## Results

There were 11 patients in the neoadjuvant group and 25 in the non-neoadjuvaunt group. The demographics between the two groups are generally comparable (Table 1). At presentation, patients who received neoadjuvant therapy had significantly larger tumour (median size > 10 cm, 33 vs. 0%, *p* = 0.04) and lower mitotic count (> 10 per 50 HPF, 14 vs. 70%, *p* = 0.03) as compared to patients who did not receive neoadjuvant therapy.

**Table 1:** Demographics of non-neoadjuvant and neoadjuvant group

Results	Non-neoadjuvant group		Neoadjuvant group		*p* value
	(*n* = 25)		(*n* = 11)		
	%	(*n*)	%	(*n*)	
Age (Median year)	58	(37 to 87)	60	(32 to 76)	0.87
Female	40%	(10/25)	46%	(5/11)	0.76
Race					0.76
Chinese	84%	(21/25)	91%	(10/11)	
Malay	12%	(3/25)	9%	(1/11)	
Indian	4%	(1/25)	0%	(0/11)	
ECOG					0.18
0	71%	(5/7)	100%	(7/7)	
1	29%	(2/7)	0%	(0/7)	
≥ 2	0%	(0/7)	0%	(0/7)	
CKIT mutation					0.60
Wild type	7%	(1/14)	0%	(0/11)	
Exon 9	14%	(2/14)	9%	(1/11)	
Exon 11	79%	(11/14)	91%	(10/11)	
Size (Median cm)	6.0	(1.2 to 10.0)	7.1	(2.6 to 11.5)	0.04*
≤ 2	19%	(3/19)	0%	(0/9)	
> 2–≤ 5	26%	(5/19)	22%	(2/9)	
> 5– ≤ 10	58%	(11/19)	44%	(4/9)	
> 10	0%	(0/19)	33%	(3/9)	
Mitotic counts					0.03*
0–5 per 50 HPF	20%	(4/20)	71%	(5/7)	
6–10 per 50 HPF	10%	(2/20)	14%	(1/7)	
> 10 per 50 HPF	70%	(14/20)	14%	(1/7)	
NIH-Fletcher risk classification					0.47
Very low/low	11%	(2/19)	25%	(2/8)	
Intermediate	19%	(3/19)	25%	(2/8)	
High	74%	(14/19)	50%	(4/8)	

*ECOG* Eastern Cooperative Oncology Group

### Neoadjuvant imatinib use

The top reasons for neoadjuvant therapy were due to an unresectable tumour (55%) or high morbidity surgery (36%) (Table 2). Most patients received 8.8 months (median months 4.5–33.9) of neoadjuvant imatinib at 400 mg or 300 mg (73 vs. 27%). Based on RECIST, majority of patients had at least a partial response to neoadjuvant therapy (82%) resulting in median size reduction of 2.7 cm or 39% size reduction.

**Table 2:** Descriptive analysis of neoadjuvant group

	Neoadjuvant group	
	(*n* = 11)	
Results	%	(*n*)
Reason for initiation		
Unresectable	55%	(6/11)
High morbidity surgery	36%	(4/11)
Does of imatinib		
400 mg	73%	(8/11)
300 mg	27%	(3/11)
Duration (Median months)	8.8	(4.5 to 33.9)
Response to imatinib		
Complete response	9%	(1/11)
Partial response	82%	(9/11)
No response	9%	(1/11)
Progressive disease	0%	(0/11)
Post-neoadjuvant size (Median cm)	3.6	(2.6 to 11.0)
Size reduction (Median cm, %)	2.7, 39%	(0–6.1, 0–61%)

Surgical Outcomes after Neoadjuvant Gleevec.

Between the neoadjuvant and non-neoadjuvant groups, both groups underwent similar rates of resection (73 vs. 88%, *p* = 0.26) and stoma creation (45 vs. 40%, *p* = 0.76), that was sphincter preserving (75 vs. 76%, *p* = 0.94), via a minimally surgical approach (26 vs. 34%, *p* = 0.55) (Table 3). Although it did not reach statistical significance, the neoadjuvant group achieved greater R0 resection (71.4 vs. 56%, *p* = 0.47) and had fewer surgical complications (9 vs. 16%, *p* = 0.58). Importantly, the non-neoadjuvant group experienced intraoperative complications (16%) consisting of ruptured or perforated tumour resulting in local and distal recurrence (50% of which were peritoneal recurrence) while the neoadjuvant group had no intraoperative complications and only developed one case of rectosigmoid fistula that was managed with a transanal excision. All patients in the non-neoadjuvant group who did not undergo surgery only had biopsies to confirm the diagnosis and subsequently defaulted follow-up. In terms of CKIT exon 9 mutation, the neoadjuvant group was able to achieve better rates of R0 resection (100 vs. 50%), with lower rates of overall recurrence (100 vs. 50%) as compared to the non-neoadjuvant group. There was no difference in terms of the use of sphincter-sparing approach (100 vs. 100%), surgical complications (0 vs. 0%) and 1-year overall survival (100 vs. 100%) between both groups. Keeping in mind the inherent limitation of running subgroup analysis in such a small population (2 in the non-neoadjuvant group and 1 in the neoadjuvant group), pooled data from various institutions of this rare tumour may better facilitate subgroup analysis to find out if the type of mutation affects the prognosis in the setting of neoadjuvant therapy.

**Table 3:** Outcome of non-neoadjuvant and neoadjuvant group

Results	Non-neoadjuvant group		Neoadjuvant group		*p* value
	(*n* = 25)		(*n* = 11)		
	%	(*n*)	%	(*n*)	
Surgical resection	88%	(22/25)	73%	(8/11)	0.26
Surgical approach					0.55
Transanal	29%	(6/21)	13%	(1/8)	
Laparoscopic	5%	(1/21)	13%	(1/8)	
Open	67%	(14/21)	75%	(6/8)	
Extend of surgery					
Sphincter sparing	76%	(16/21)	75%	(6/8)	0.94
Stoma creation	40%	(10/25)	45%	(5/11)	0.76
Extend of resection					0.47
R0		56%	(10/18)	71.4%	(5/7)
R1		44%	(8/18)	29%	(2/7)
Surgical complications	16%	(4/25)	9%	(1/11)	0.58
Ruptured tumour	50%	(2/4)	0%	(0/1)	
Perforated tumour	50%	(2/4)	0%	(0/1)	
Fistula	0%	(0/4)	100%	(1/1)	
Adjuvant therapy	40%	(10/25)	60%	(6/10)	0.28
Dose of adjuvant imatinib					0.23
400 mg	100%	(8/8)	83%	(5/6)	
300 mg	0%	(0/8)	17%	(1/6)	
Duration of imatinib	3.0	(0.3 to 6.2)	2.1	(0.3 to 2.5)	0.41
(Median year)					
Total duration of imatinib	3.1	(0.3 to 6.2)	2.0	(0.3 to 3.1)	0.40
(Median year)					
Response to imatinib					0.22
Complete response	30%	(3/10)		83%	(5/6)
Partial response		10%	(1/10)	0%	(0/6)
No response		10%	(1/10)	0%	(0/6)
Progressive disease	50%	(5/10)	17%	(1/6)	

Adjuvant Therapy Post-resection.

Although both the neoadjuvant and non-neoadjuvant therapy groups underwent adjuvant therapy (60 vs. 40%, *p* = 0.28), the neoadjuvant group underwent a shorter duration of adjuvant therapy (2.1 vs. 3.0 years, *p* = 0.41) and achieved greater rates of complete response (83 vs. 30%, *p* = 0.22) based on RECIST, though it did not achieve statistical significance (Table 3). In addition, the total duration of imatinib (accounting for both neoadjuvant and adjuvant) were comparable between both groups (2.0 vs. 3.1 median years, *p* = 0.40).

### Recurrence and survival outcomes

**Fig. 1:**
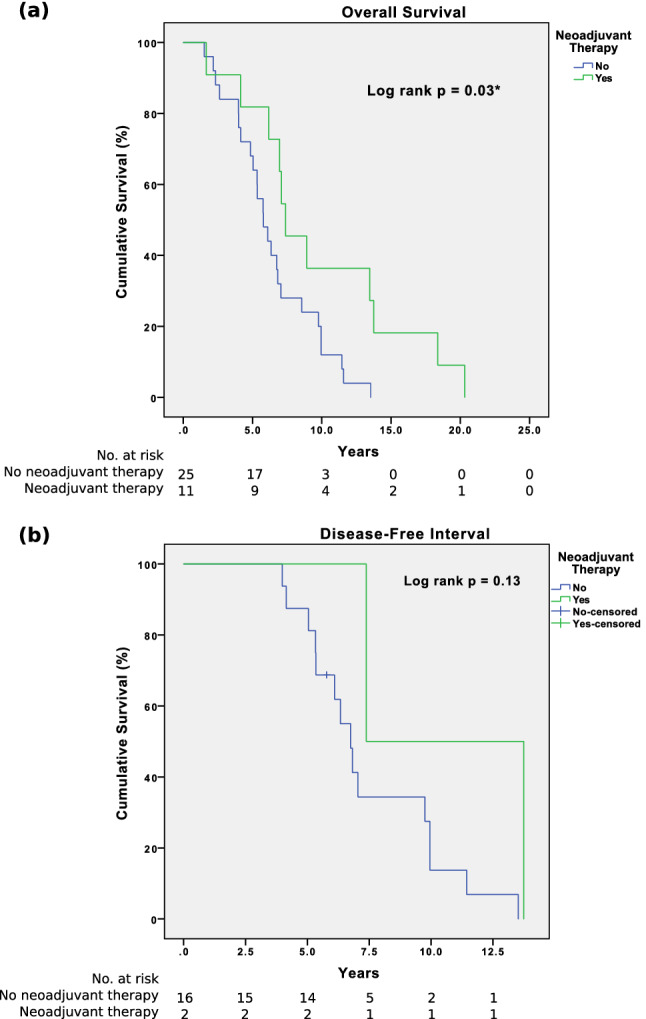
Kaplan–Meier **a** overall survival (*p* = 0.03) and **b** disease-free interval (*p* = 0.13) between neoadjuvant and non-neoadjuvant groups

The recurrence rate for the neoadjuvant group was statistically lower than the non-neoadjuvant group (18 vs. 64%, *p* = 0.01), with the former having a lower rate of local recurrence (0 vs. 69%, *p* = 0.04) (Table 4). There were no cases of local recurrence in the neoadjuvant group while the median time to local recurrence in the non-neoadjuvant group was 3.9 years. On the other hand, the median time to distant recurrence in the neoadjuvant and non-neoadjuvant groups were comparable (3.3 vs. 3.6 years, *p* = 0.63). For both groups, all recurrence cases did not undergo surgical treatment. Instead, adjuvant chemotherapy was restarted in 75% (12/16) and 100% (2/2) in the neoadjuvant group. Unfortunately, 7 patients are still currently undergoing therapy and we are unable to run further analysis to evaluate the efficacy of the treatment. There were 2 neoadjuvant cases that experienced tumour recurrence, specifically distal recurrence involving the liver and peritoneum. Both cases had CKIT exon 11 mutation and experienced recurrence at 3.4 and 3.3 year after the initial surgery, respectively. Both cases are still undergoing imatinib therapy. The neoadjuvant group had a longer OS (7.4 vs. 5.7 years, *p* = 0.03) as compared to the non-neoadjuvant group (Fig. 1). Although it did not achieve statistically significance, the early 1-year DFS (100 vs. 94%, *p* = 0.72) and OS (100 vs. 100%) and 3-year DFS (100 vs. 75%, *p* = 0.42) and OS (91 vs. 84%, *p* = 0.59) between the neoadjuvant and non-neoadjuvant groups were similarly high. At the 5-year mark, the neoadjuvant group had poorer DFS (0 vs. 31%, *p* = 0.35), but had better OS (82 vs. 68%, *p* = 0.39) as compared to the non-neoadjuvant group.

**Table 4:** Survival and recurrence data of non-neoadjuvant and neoadjuvant group

Results	Non-neoadjuvant group		Neoadjuvant group		*p* value
	(*n* = 25)		(*n* = 11)		
	%	(*n*)	%	(*n*)	
Recurrence	64%	(16/25)	18%	(2/11)	0.01*
Local	69%	(11/16)	0%	(0/2)	0.04*
Distal	63%	(10/16)	100%	(2/2)	0.29
Site of distal metastasis					0.30
Liver	50%	(5/10)	50%	(1/2)	
Peritoneal	10%	(1/10)	50%	(1/2)	
Multiple	40%	(4/10)	0%	(0/2)	
Time to local recurrence (Median year)	3.9	(0.9 to 15.1)	-		-
Time to distal recurrence (Median year)	3.6	(0.9 to 10.4)	3.3	(3.3 to 3.4)	0.63
1-year disease-free interval	94%	(15/16)	100%	(2/2)	0.72
3-year disease-free interval	75%	(12/16)	100%	(2/2)	0.42
5-year disease-free interval	31%	(5/16)	0%	(0/2)	0.35
Overall survival	5.7	(1.5 to 13.5)	7.4	(1.7 to 20.3)	0.03*
(Median year)					
1-year overall survival	100%	(25/25)	100%	(11/11)	-
3-year overall survival	84%	(21/25)	91%	(10/11)	0.59
5-year overall survival	68%	(17/25)	82%	(9/11)	0.39

## Discussion

The applicability of imatinib for the treatment of GIST in the neoadjuvant setting is an area of huge research interest. The National Comprehensive Cancer Network recommends neoadjuvant systemic therapy for borderline-resectable or oligometastatic metastatic GIST on a case-to-case basis [11]. It recommends that preoperative imatinib treatment should be considered if abdominoperineal resection or multivisceral resection is necessary to achieve a negative resection margin. On the other hand, the European Society for Medical Oncology guidelines recommend neoadjuvant therapy if R0 resection is feasible, achieved by less mutilating surgery, safer surgical procedure, lesser blood loss and lower risk of tumour rupture [12].

However, the inclusion criteria and optimal duration for neoadjuvant therapy remain poorly defined. Some studies have recommended a tumour size cut-off of 5 cm to initiate preoperative imatinib therapy while other studies have recommended initiation in high-risk tumours [3, 8, 13–16]. RTOG 0132/ACRIN 6665 study recommended duration of 8–12 weeks of preoperative therapy while other smaller studies saw benefit for treatment duration of 6–15 months [3, 14, 15, 17–20]. Besides having no definitive information on the length of neoadjuvant therapy, there is also a paucity of data on the ideal clinical endpoint. Based on the limited data that were published, a final tumour size of 1.8–6.9 cm after 19–63% reduction conferred surgical and/or survival benefit [14, 16, 20–26].

Our study’s findings may provide further insights insofar as to aid the future development of a neoadjuvant therapy guidelines. We found that patients who received neoadjuvant therapy had larger tumours (7.1 vs. 6.0 cm, *p* = 0.04) with lower mitotic count (71 vs. 20% of 0–5 mitotic count per 50 HPF, *p* = 0.03) as compared to those who had no neoadjuvant therapy. A duration of 8.8 months of neoadjuvant therapy was useful in downsizing tumour to a median size of 3.6 cm after a 2.7 cm or 39% size reduction to improve resectability to the extent of achieving R0 resection, with low complications and improvements in sphincter-sparing rates. Neoadjuvant TKI improved the eligibility of sphincter-sparing procedures to the level that was comparable to the non-neoadjuvant group for patients who were considered to be surgically inoperable or challenging. Notably, neoadjuvant therapy generally conferred protection from local recurrence and improved OS regardless of tumour size and tumour risk stratification.

In reported literature, the mean size of GIST ranges from 10 to 13 cm with those greater than 5 cm more likely to be symptomatic (e.g. bleeding and abdominal pain) [1, 27]. On the other hand, due to the paucity of space in the pelvis, rectal GIST tends to become symptomatic at a smaller size and present with a mean size of 4.8 cm [15]. The high mitotic activity in rectal GISTs remains the main determinant factor in their risk categorization (not their size). In addition, a predominance of rectal GISTs has KIT exon 11 deletions, which has been linked to adverse outcomes [28]. Interestingly, we found out that the patients who had neoadjuvant therapy had initial lower mitotic count, which suggested that they might be inherently less aggressive as compared to the non-neoadjuvant as evident by lower high-risk tumours by NIH-Fletcher risk classification (50 vs. 74%, *p* = 0.47).

Surgical resection with negative margins represents the most effective chance of long-term survival in patients with non-metastatic, low-risk GISTs [12]. Likewise, the mainstay of treatment for rectal GISTs is complete resection with clear margins without mandatory lymph node dissection (lymphatic metastases are exceedingly rare). There is good clinical outcome in general for resectable tumours but it is accompanied with a high rate of recurrence and metastasis within 2 years [6]. Owing to the rarity of rectal GIST, no standard surgical treatment exists for tumours localized in the rectum. Conventional surgical methods to treat rectal GISTs include abdominoperineal resection, total exenteration and anterior resection. A large proportion of rectal GIST is also accessible to local excision via transanal, transsacral or transvaginal approaches. Local excision has been reported to decrease the risk of peritoneal recurrence as it preserves the integrity of the peritoneum at the rectosigmoid junction. The choice of procedure depends on the location; tumour size, extent and the probability in achieving clear margins. In anatomical challenging cases due to tumour size, location and relationship with pelvic structures, abdominoperineal or multivisceral resections may be performed, but raises the problem of sphincter preservation. Extensive surgery may result in considerable functional morbidity. Therefore, it is important to consider the balance of radical resection with the preservation of the anal, urinary and sexual function in the surgical treatment of rectal GIST. Generally, wide margins are not necessary if a clear resection margin is obtained. Although extended surgery achieves more R0 resection compared with conservative surgery, the rate of tumour recurrence is comparable between both groups as illustrated by the study by Khalifa et al. who reported no difference in survival rates between local excision and abdominoperineal resection for patients with rectal GISTs [29].

Adjuvant imatinib following surgery is well established for high risk GISTs. According to two independent high-impact studies conducted by Joensuu et al. and Mietinen et al., the initiation of adjuvant imatinib for high-risk rectal GIST to decrease the risk of recurrence and metastasis is recommended [30, 31]. In addition, incomplete surgical resection or intraoperative tumour rupture are also common reasons for patients to start adjuvant imatinib postoperatively. Unlike the duration of neoadjuvant therapy, the optimal duration for adjuvant therapy has been defined as 3 years [31]. The need for adjuvant therapy for patients with low-risk GIST who respond to neoadjuvant imatinib and have undergone complete surgical resection remains unanswered.

In this study, we found that neoadjuvant therapy enabled a modification in tumour size and/or density to permit the utility of minimally invasive sphincter-sparing surgery in 36% of our patients. It is important to note that resistance to relevant secondary mutations may occur after neoadjuvant therapy and hence the duration of treatment should be as short as possible to facilitate timely complete resection [32]. Neoadjuvant therapy can be continued until the regression of the tumour size or metabolic activity reaches a plateau phase as the development of secondary KIT mutations is common in protracted treatment duration. As a result, Haller et al. advised to continue therapy until stagnation of tumour shrinkage is obtained, which signifies that maximum effect has been achieved [20]. Given the fact that most of the response to imatinib occurs within 6 months of therapy, surgery should be done promptly at approximately 6 months of therapy before the development or selection of clones with secondary mutations. Therefore, mutational analysis is crucial to distinguish the resistant genotypes that will not respond to targeted therapy in order to avoid delay of surgical excision. Mutations in exon 11 of KIT have been generally associated with tumours that are imatinib-sensitive while tumours with mutation in exon 9 tend to be resistant to imatinib [33]. In general, imatinib can be safely discontinued 2–3 days prior to surgery and can restart immediately once patient recovers from the surgery.

Different imaging modalities are used to monitor the response after systemic therapy. The most commonly used is RECIST [10]. However, RECIST has some inherent limitations as it focuses mainly on size reduction. The Choi response criteria are purported to be a better alternative as it is based on both a decrease in tumour size and tumour density on contrast-enhanced CT leading to better prediction of time to tumour progression [34]. In cases with imaging ambiguity, PET can complement CT scans [35]. PET scan allows quantitative comparison of tumour metabolism different time points. GIST responsiveness to imatinib can be seen as early as 1 week after initiation of therapy and more than 25% reduction in metabolism is considered to be an adequate response to systemic therapy. In addition, PET scan is especially useful in the neoadjuvant setting as it may affect clinical decision to proceed with surgical resection or continuing systemic therapy as it can detect early evaluation of neoadjuvant imatinib response better than CT scans [36].

Given the complexity and rarity of rectal GIST, a multidisciplinary approach with a team of surgical oncologists, medical oncologists, radiologists and pathologists with expertise in sarcomas is imperative to provide tailored management for the patient. Recurrence can develop up to 10–15 years after surgical therapy and therefore requires long-term follow-up.

Inferences drawn from our study should be tempered by the fact that it is based on a single-centre retrospective study. The retrospective nature of this study renders it susceptible to selection bias. Given that this is a single centre study looking at a rare condition, it is compounded by the low incidence of rectal GIST resulting in our study sample to be small and hence prone to type 2 errors. Also, there are no optimal cut-off values for initiation and termination for neoadjuvant therapy. Future studies in the area of inflammatory indices may look into optimal cut-offs for patients. In addition, patients selected for neoadjuvant imatinib and the selection of surgical technique were also not properly defined. The lack of these selection criteria muddles the conclusion of our study. Furthermore, in Tables 1 and 3, 10 of 17 patients in the non-neoadjuvant group with intermediate and high-risk tumours received adjuvant therapy, whereas all 6 patients in the neoadjuvant group with intermediate and high-risk tumours received adjuvant therapy. This might lead to bias in the survival difference seen between the two arms.

Although this study is small in number and respective in designs, it adds to the increasing evidence to support the use of neoadjuvant imatinib therapy for the treatment of rectal GIST. In selected cases that are not amenable with surgical resection at the onset, administration of neoadjuvant imatinib of approximately 9 months has the benefit of reducing the tumour size by 39% to improve resectability to achieve R0 resection with low complication rates using sphincter-sparing techniques. In addition, it confers protection from local recurrence and improved OS regardless of tumour size and tumour risk stratification. The benefits of neoadjuvant imatinib also extend to GIST of other sites. Neoadjuvant imatinib has been shown to downsize tumour to achieve complete surgical resection in gastroesophageal GIST [37–41]. The landmark paper from EORTC STBSG sarcoma centres had consensual results for upper gastrointestinal GIST, which noted a median overall survival of 8.6 years in the neoadjuvant group [42].

In conclusion, our study found that a therapeutic strategy that combines neoadjuvant imatinib therapy with surgery should be considered for patients with rectal GISTs that are large, marginally resectable or unresectable or have a close relationship with vital pelvic structures, such as anal sphincters or nerves. Neoadjuvant imatinib may potentially increase the proportion of patients able to undergo curative conservative surgery rather than the more morbid extended resection.

## References

[1] Valsangkar N, Sehdev A, Misra S, Zimmers TA, O'Neil BH, Koniaris LG (2015). Current management of gastrointestinal stromal tumors: Surgery, current biomarkers, mutations, and therapy. Surgery.

[2] Gervaz P, Huber O, Morel P (2009). Surgical management of gastrointestinal stromal tumours. Br J Surg.

[3] Liu H, Yan Z, Liao G, Yin H (2014). Treatment strategy of rectal gastrointestinal stromal tumor (GIST). J Surg Oncol.

[4] Eisenberg BL, Pipas JM (2012). Gastrointestinal stromal tumor–background, pathology, treatment. Hematol Oncol Clin North Am.

[5] von Mehren M (2016). Management of gastrointestinal stromal tumors. Surg Clin North Am.

[6] Yasui M, Tsujinaka T, Mori M, Takahashi T, Nakashima Y, Nishida T, Kinki GSG (2017). Characteristics and prognosis of rectal gastrointestinal stromal tumors: an analysis of registry data. Surg Today.

[7] Demetri GD, Von Mehren M, Blanke CD, Van den Abbeele AD, Eisenberg B, Roberts PJ, Heinrich MC, Tuveson DA, Singer S, Janicek M, Fletcher JA, Silverman SG, Silberman SL, Capdeville R, Kiese B, Peng B, Dimitrijevic S, Druker BJ, Corless C, Fletcher CD, Joensuu H (2002). Efficacy and safety of imatinib mesylate in advanced gastrointestinal stromal tumors. N Engl J Med.

[8] Wilkinson MJ, Fitzgerald JE, Strauss DC, Hayes AJ, Thomas JM, Messiou C, Fisher C, Benson C, Tekkis PP, Judson I (2015). Surgical treatment of gastrointestinal stromal tumour of the rectum in the era of imatinib. Br J Surg.

[9] Fletcher CD, Berman JJ, Corless C, Gorstein F, Lasota J, Longley BJ, Miettinen M, O'Leary TJ, Remotti H, Rubin BP, Shmookler B, Sobin LH, Weiss SW (2002). Diagnosis of gastrointestinal stromal tumors: a consensus approach. Hum Pathol.

[10] Eisenhauer EA, Therasse P, Bogaerts J, Schwartz LH, Sargent D, Ford R, Dancey J, Arbuck S, Gwyther S, Mooney M, Rubinstein L, Shankar L, Dodd L, Kaplan R, Lacombe D, Verweij J (2009). New response evaluation criteria in solid tumours: revised RECIST guideline (version 1.1). Eur J Cancer.

[11] von Mehren M, Randall RL, Benjamin RS, Boles S, Bui MM, Ganjoo KN, George S, Gonzalez RJ, Heslin MJ, Kane JM, Keedy V, Kim E, Koon H, Mayerson J, McCarter M, McGarry SV, Meyer C, Morris ZS, O'Donnell RJ, Pappo AS, Paz IB, Petersen IA, Pfeifer JD, Riedel RF, Ruo B, Schuetze S, Tap WD, Wayne JD, Bergman MA, Scavone JL (2018). Soft tissue sarcoma, version 2.2018, NCCN clinical practice guidelines in oncology. J Natl Compr Canc Netw.

[12] Casali PG, Abecassis N, Bauer S, Biagini R, Bielack S, Bonvalot S, Boukovinas I, Bovee J, Brodowicz T, Broto JM, Buonadonna A, De Alava E, Dei Tos AP, Del Muro XG, Dileo P, Eriksson M, Fedenko A, Ferraresi V, Ferrari A, Ferrari S, Frezza AM, Gasperoni S, Gelderblom H, Gil T, Grignani G, Gronchi A, Haas RL, Hannu A, Hassan B, Hohenberger P, Issels R, Joensuu H, Jones RL, Judson I, Jutte P, Kaal S, Kasper B, Kopeckova K, Krakorova DA, Le Cesne A, Lugowska I, Merimsky O, Montemurro M, Pantaleo MA, Piana R, Picci P, Piperno-Neumann S, Pousa AL, Reichardt P, Robinson MH, Rutkowski P, Safwat AA, Schoffski P, Sleijfer S, Stacchiotti S, Sundby Hall K, Unk M, Van Coevorden F, Van der Graaf W, Whelan J, Wardelmann E, Zaikova O, Blay JY, Committee EG, Euracan (2018). Gastrointestinal stromal tumours: ESMO-EURACAN Clinical Practice Guidelines for diagnosis, treatment and follow-up. Ann Oncol.

[13] Hawkins AT, Wells KO, Krishnamurty DM, Hunt SR, Mutch MG, Glasgow SC, Wise PE, Silviera ML (2017). Preoperative chemotherapy and survival for large anorectal gastrointestinal stromal tumors: a national analysis of 333 cases. Ann Surg Oncol.

[14] Tielen R, Verhoef C, van Coevorden F, Reyners AK, van der Graaf WT, Bonenkamp JJ, van Etten B, de Wilt JH (2013). Surgical management of rectal gastrointestinal stromal tumors. J Surg Oncol.

[15] Jakob JMC, Ronellenfitsch U, Wardelmann E, Negri T, Gronchi A, Hohenberger P (2013). Gastrointestinal stromal tumor of the rectum—results of surgical and multimodality therapy in the era of imatinib. Ann Surg Oncol.

[16] Ueki T, Nagayoshi K, Manabe T, Maeyama R, Yokomizo A, Yamamoto H, Oda Y, Tanaka M (2014). Laparoscopic en bloc excision of gastrointestinal stromal tumors of the rectum after neoadjuvant imatinib therapy: anteriorly extended intersphincteric resection combined with partial resection of the prostate. Tech Coloproctol.

[17] Eisenberg BL, Harris J, Blanke CD, Demetri GD, Heinrich MC, Watson JC, Hoffman JP, Okuno S, Kane JM, von Mehren M (2009). Phase II trial of neoadjuvant/adjuvant imatinib mesylate (IM) for advanced primary and metastatic/recurrent operable gastrointestinal stromal tumor (GIST): early results of RTOG 0132/ACRIN 6665. J Surg Oncol.

[18] Huynh TKMP, Cassier P, Bouché O, Lardière-Deguelte S, Adenis A, André T, Mancini J, Collard O, Montemurro M, Bompas E, Rios M, Isambert N, Cupissol D, Blay JY, Duffaud F (2014). Primary localized rectal/pararectal gastrointestinal stromal tumor—results of surgical and multimodal therapy from the French Sarcoma group. BMC Cancer.

[19] Zanwar S, Ostwal V, Sahu A, Jain D, Ramaswamy A, Saklani A, Ramadwar M, Shetty N, Shrikande SV (2016). Rectal GIST—outcomes and viewpoint from a tertiary cancer center. Indian J Gastroenterol.

[20] Haller F, Detken S, Schulten HJ, Happel N, Gunawan B, Kuhlgatz J, Fuzesi L (2007). Surgical management after neoadjuvant imatinib therapy in gastrointestinal stromal tumours (GISTs) with respect to imatinib resistance caused by secondary KIT mutations. Ann Surg Oncol.

[21] Fujimoto Y, Akiyoshi T, Konishi T, Nagayama S, Fukunaga Y, Ueno M (2014). Laparoscopic sphincter-preserving surgery (intersphincteric resection) after neoadjuvant imatinib treatment for gastrointestinal stromal tumor (GIST) of the rectum. Int J Colorectal Dis.

[22] Ebihara Y, Okushiba S, Kawarada Y, Kitashiro S, Katoh H, Kondo S (2008). Neoadjuvant imatinib in a gastrointestinal stromal tumor of the rectum: report of a case. Surg Today.

[23] Kaneko M, Nozawa H, Emoto S, Murono K, Sasaki K, Otani K, Nishikawa T, Tanaka T, Kiyomatsu T, Hata K, Kawai K, Watanabe T (2017). Neoadjuvant Imatinib therapy followed by intersphincteric resection for low rectal gastrointestinal stromal tumors. Anticancer Res.

[24] Kyo K, Azuma M, Okamoto K, Nishiyama M, Shimamura T, Maema A, Kanamaru H, Shirakawa M, Nakamura T, Shinmura K, Koda K, Yokoyama H (2016). Neoadjuvant imatinib treatment and laparoscopic anus-preserving surgery for a large gastrointestinal stromal tumor of the rectum. World J Surg Oncol.

[25] Kramp KH, Omer MG, Schoffski P, d'Hoore A (2015). Sphincter sparing resection of a large obstructive distal rectal gastrointestinal stromal tumour after neoadjuvant therapy with imatinib (Glivec). BMJ Case Rep.

[26] Wang J-P, Wang T, Huang M-J, Wang L, Kang L, Wu X-J (2011). The Role of neoadjuvant imatinib mesylate therapy in sphincter-preserving procedures for anorectal gastrointestinal stromal tumor. Am J Clin Oncol.

[27] Kukar M, Kapil A, Papenfuss W, Groman A, Grobmyer SR, Hochwald SN (2015). Gastrointestinal stromal tumors (GISTs) at uncommon locations: a large population based analysis. J Surg Oncol.

[28] Ricci RMM, Cenci T, Antinori A, Cassano A, Larocca LM (2016). Case of rectal GI stromal tumor demonstrating that KIT and PDGFRA mutations are not always mutually exclusive. J Clin Oncol.

[29] Khalifa AABW, Rao VK, Williams MJ (1986). Leiomyosarcoma of the rectum. Report of a case and review of the literature. Dis Colon Rectum.

[30] Miettinen MFM, Sarlomo-Rikala M, Burke A, Sobin LH, Lasota J (2001). Gastrointestinal stromal tumors, intramural leiomyomas, and leiomyosarcomas in the rectum and anus—a clinicopathologic, immunohistochemical, and molecular genetic study of 144 cases. Am J Surg Pathol.

[31] Joensuu HEM, Sundby Hall K, Hartmann JT, Pink D, Schütte J, Ramadori G, Hohenberger P, Duyster J, Al-Batran SE, Schlemmer M, Bauer S, Wardelmann E, Sarlomo-Rikala M, Nilsson B, Sihto H, Monge OR, Bono P, Kallio R, Vehtari A, Leinonen M, Alvegård T, Reichardt P (2012). One vs three years of adjuvant imatinib for operable gastrointestinal stromal tumor—a randomized trial. JAMA.

[32] Wardelmann E (2006). Polyclonal evolution of multiple secondary KIT mutations in gastrointestinal stromal tumors under treatment with imatinib mesylate. Clin Cancer Res.

[33] Vadakara J, von Mehren M (2013). Gastrointestinal stromal tumors: management of metastatic disease and emerging therapies. Hematol Oncol Clin North Am.

[34] Benjamin RS, Choi H, Macapinlac HA, Burgess MA, Patel SR, Chen LL, Podoloff DA, Charnsangavej C (2007). We should desist using RECIST, at least in GIST. J Clin Oncol.

[35] Van den Abbeele AD (2008). The lessons of GIST–PET and PET/CT: a new paradigm for imaging. Oncologist.

[36] Farag S, Geus-Oei L-F, van der Graaf WT, van Coevorden F, Grunhagen D, Reyners AKL, Boonstra PA, Desar I, Gelderblom H, Steeghs N (2018). Early evaluation of response using 18F-FDG PET influences management in gastrointestinal stromal tumor patients treated with neoadjuvant imatinib. J Nucl Med.

[37] Verweij J, Casali PG, Zalcberg J, LeCesne A, Reichardt P, Blay J-Y, Issels R, van Oosterom A, Hogendoorn PCW, Van Glabbeke M, Bertulli R, Judson I (2004). Progression-free survival in gastrointestinal stromal tumours with high-dose imatinib: randomised trial. Lancet.

[38] Tirumani SH, Shinagare AB, Jagannathan JP, Krajewski KM, Ramaiya NH, Raut CP (2014). Radiologic assessment of earliest, best, and plateau response of gastrointestinal stromal tumors to neoadjuvant imatinib prior to successful surgical resection. Eur J Surg Oncol (EJSO).

[39] Senichiro Yanagawa KT, Suzuki T, Tokumoto N, Arihiro K, Ohdan H (2014). A large esophageal gastrointestinal stromal tumor that was successfully resected after neoadjuvant imatinib treatment—case report. World J Surg Oncol.

[40] Neofytou K, Costa Neves M, Giakoustidis A, Benson C, Mudan S (2015). Effective downsizing of a large oesophageal gastrointestinal stromal tumour with neoadjuvant imatinib enabling an uncomplicated and without tumour rupture laparoscopic-assisted ivor-lewis oesophagectomy. Case Rep Oncol Med.

[41] Asif S, Gupta N, Gupta G, Mehta A, Singh S (2016). effective downsizing of a gastroesophageal GIST using neoadjuvant imatinib mesylate: a case report. J Gastrointest Cancer.

[42] Rutkowski P, Gronchi A, Hohenberger P, Bonvalot S, Schöffski P, Bauer S, Fumagalli E, Nyckowski P, Nguyen B-P, Kerst JM, Fiore M, Bylina E, Hoiczyk M, Cats A, Casali PG, Le Cesne A, Treckmann J, Stoeckle E, de Wilt JHW, Sleijfer S, Tielen R, van der Graaf W, Verhoef C, van Coevorden F (2013). Neoadjuvant imatinib in locally advanced gastrointestinal stromal tumors (GIST): the EORTC STBSG experience. Ann Surg Oncol.

